# Direct percutaneous access of the thoracic duct in a neonate as curative treatment of a high-output life-threatening chylothorax due to thrombotic occlusion of the thoracic duct–venous junction

**DOI:** 10.1007/s00247-025-06412-1

**Published:** 2025-10-02

**Authors:** Daniel Kronenberg, Hitendu Dave, Oliver Kretschmar, Alessandra Bosch, Janet Kelly-Geyer, Christian Johannes Kellenberger, Ralph Gnannt

**Affiliations:** 1 Department of Pediatric Diagnostic Imaging and Intervention, University Children’s Hospital Zurich, Lenggstrasse 30, 8008 Zurich, Switzerland; 2 Department of Pediatric Cardiac Surgery, University Children’s Hospital Zurich, Zurich, Switzerland; 3 Department of Pediatric Cardiology, University Children’s Hospital Zurich, Zurich, Switzerland; 4 Department of Pediatric Hematology, University Children’s Hospital Zurich, Zurich, Switzerland; 5 Department of Pediatric Intensive Care and Neonatology, University Children’s Hospital Zurich, Zurich, Switzerland

**Keywords:** Chylothorax, Congenital heart disease, Neonate, Recanalization, Thoracic duct obstruction, Ultrasound

## Abstract

A life-threatening chylothorax developed in a female neonate after corrective surgery of d-transposition of the great arteries complicated by extensive postoperative thrombosis of the superior vena cava distribution, including at the thoracic duct–venous junction. Emergent percutaneous catheter intervention for thrombus aspiration and transluminal angioplasty was required. Despite therapeutic heparinization, thrombosis persisted. Curative image-guided treatment was twofold: first, the occluded thoracic duct was punctured under ultrasound guidance; then, the thrombus at the thoracic duct–venous junction was mobilized using the Seldinger-technique. Additionally, a venous catheter was placed with the tip at the thoracic duct–venous junction, and local low-dose thrombolysis was administered. This case shows that it is possible to percutaneously access the thoracic duct by direct puncture in a neonate with ultrasound guidance.

## Introduction

The lymphatic system plays an important role in the interstitial fluid balance [1]. The lymphatic fluid drains back into the venous system via the thoracic duct at the thoracic duct–venous junction, which is usually located at the junction of the left subclavian and left internal jugular vein. Over 80% of this fluid, the chyle, originates from the liver and intestines [1]. A disruption or blockage of chylous flow can cause a chylothorax/chyleascites, which can cause major morbidity and mortality in neonates [2]. Guevara CJ et al. demonstrated that direct percutaneous access of the thoracic duct is feasible and applicable to multiple treatment modalities [3] .

## Case report

Informed consent was obtained from the parents for publishing the case report. A female neonate with antenatally diagnosed d-transposition of the great arteries with a perimembranous ventricular septal defect was transferred to our institution for treatment and corrective surgery on day 1 of life. A balloon atrial septostomy (Rashkind Procedure) was performed on day 2 of life due to inadequate mixing. On day 5 of life, an arterial switch operation and a ventricular septal defect closure with the LeCompte maneuver were performed, and a central venous catheter was placed in the right internal jugular vein the following day. On day 15 of life, she developed superior vena cava (SVC) syndrome and extensive venous thrombosis in the SVC distribution, despite postoperative prophylactic anticoagulation with unfractionated heparin. The etiology remains unclear; high venous pressure from diastolic dysfunction has potentially contributed.

**Fig. 1:**
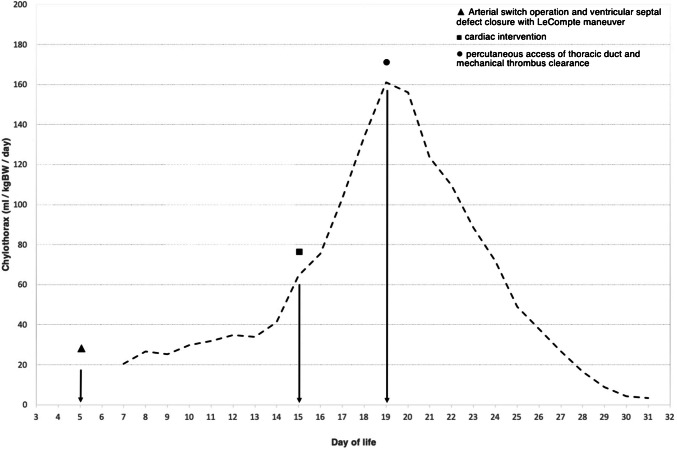
Chylothorax output over time in a 3-kg female neonate. Arterial switch operation and ventricular septal defect closure with the LeCompte maneuver (*triangle*). The cardiology team performs a percutaneous catheter intervention to aspirate thrombus in both the brachiocephalic veins and the superior vena cava, and performs transluminal angioplasty (*box*). The interventional radiology team percutaneously accesses the thoracic duct and mechanically clears it of thrombus (*circle*), with subsequent regression of chylothorax output. *ml/kgBW/day*, milliliter per kilogram body weight per day

There was occlusive thrombosis of the right internal jugular vein, the superior vena cava, and the right and left brachiocephalic veins, and in the confluence of the left internal jugular vein with the left subclavian vein in the left venous angle. Due to acute SVC syndrome, the cardiology team performed an emergent percutaneous catheter intervention on day 15 of life, during which thrombus in both the brachiocephalic veins and the superior vena cava was aspirated, and transluminal angioplasty was performed (Fig. 1). As a result, blood flow through these vessels was re-established.

After the SVC thrombosis, the 3-kg neonate also developed a high-output chylothorax with loss of up to 199 ml/kg body weight of chyle per day on day 19 of life, 4 days after thrombus aspiration and transluminal angioplasty. Chylous loss data from thoracic and pericardial drains were summed to calculate the total daily chylous losses as these compartments were connected after the arterial switch operation (Fig. 1). The high-output chylothorax was associated with progressive anasarca. A follow-up ultrasound (Aplio i800, Canon Medical Systems, Otawara, Japan) on day 19 of life demonstrated a blood clot at the left venous angle. This thrombus extended a few millimeters into a fully obstructed tubular structure without flow in duplex ultrasound, which inserted into the left venous angle. This obstructed structure was suspected to be the dilated thoracic duct.

As a relevant additional comorbidity, the cerebral ultrasound on day 15 showed a small subacute posthemorrhagic/-ischemic lesion at the junction of white matter and basal ganglia on the left side, most likely a perioperative complication. This cerebral lesion was considered to increase the potential risk of an intracranial bleed if a systemic lysis were performed. An emergency multidisciplinary meeting (pediatric intensive care, cardiac surgery, cardiology, hematology, neurology, and radiology) was held to discuss potential treatment options for this unstable neonate. After weighing benefits and risks, it was decided to perform percutaneous access of the thoracic duct with the aim of manually disrupting the thrombus and also to insert a catheter with the tip at the venous junction to enable a localized thrombolysis.

**Fig. 2:**
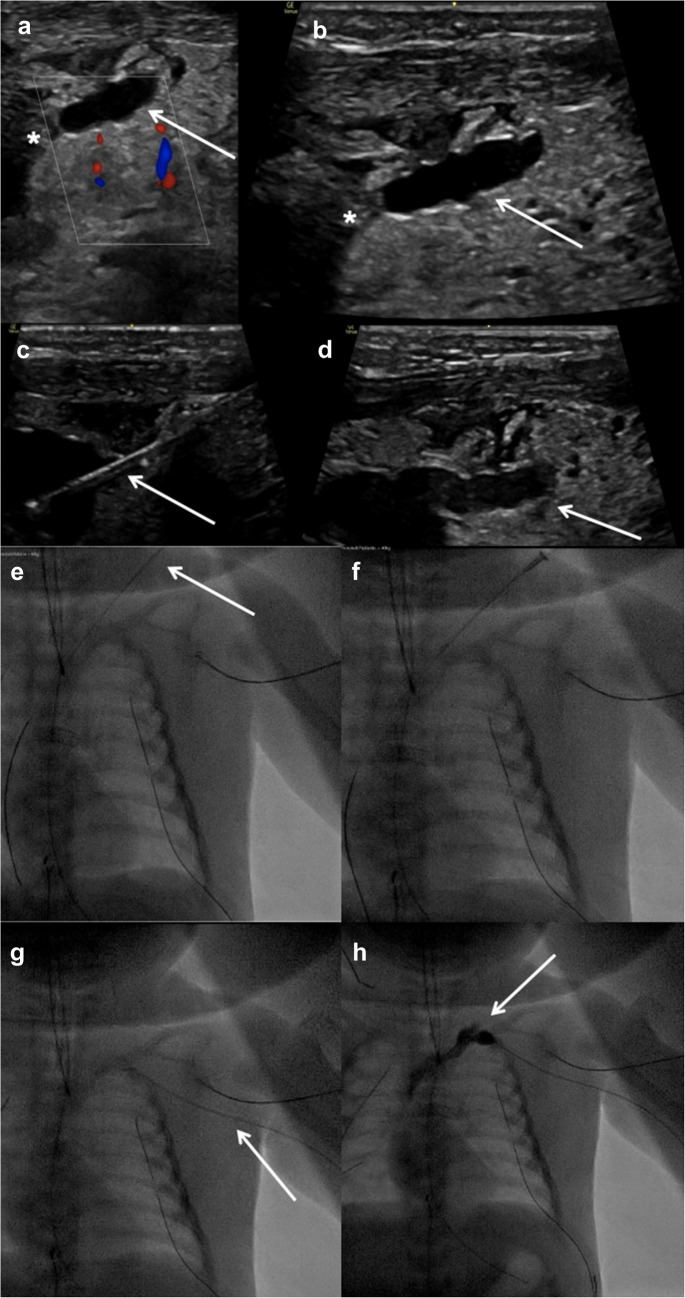
A 3-kg female neonate with high-output chylothorax. **a**-**d** Longitudinal ultrasound images demonstrate a thrombus (*asterisk*) occluding the outflow of the dilated thoracic duct (*arrow*) at the thoracic duct–venous junction with (**a**) and without (**b**) color Doppler. Under ultrasound guidance, the thoracic duct was punctured via the left neck with a 22-G peripheral venous catheter and a 0.014-in. floppy guidewire (*arrow* in **c**) was advanced through the needle and the occluding thrombus. The thoracic duct was cleared of thrombus at its venous junction (*arrow* in **d**). **e**–**f** Cropped anteroposterior fluoroscopic chest radiographs demonstrating the procedure. After removal of the peripheral venous catheter from the guidewire (*arrow* in **e**), the access tract was sequentially dilated with a 20-G cannula followed by a 14-G peripheral vein catheter using the Seldinger technique. Mechanical thrombus mobilization is demonstrated on the anteroposterior fluoroscopic chest radiograph (**f**). A catheter (*arrow* in **g**) was introduced via the left brachial vein, with its tip positioned at the thoracic duct–venous junction demonstrated on the fluoroscopic chest radiograph. Correct catheter placement is confirmed after contrast administration, with opacification of the left brachiocephalic vein and superior vena cava on fluoroscopic chest radiograph (**h**). Minimal opacification of the thoracic duct (*arrow* in **h**) is noted, consistent with backflow from the venous junction into the duct

Under sonographic guidance (Venue Go, GE HealthCare, Chicago, IL), an interventional radiology team accessed the thoracic duct percutaneously with a peripheral venous catheter (Venflon, Becton Dickinson, Sandy, UT) (Fig. 2). A mixture of blood and serous-milky fluid was drained, consistent with a catheter correctly positioned in the thoracic duct. A 0.014-in. floppy guidewire (Nitrex, ev3, Plymouth, MN) was advanced into the right atrium through the thrombus (Fig. 2). Sequential dilatation of the thoracic duct junction over the wire was performed with peripheral venous catheters up to 12-G, and the thrombus was mobilized (Fig. 2). At the end of the intervention, patency of the thoracic duct and orthograde flow of chyle into the venous confluence was demonstrated sonographically (Fig. 2). Thereafter, a venous catheter (Vascu-PICC, Medcomp, Yuma, AZ) was placed using combined sonographic and fluoroscopic guidance (Azurion Biplane, Philips Healthcare, Best, Netherlands) via the left brachial vein, with the tip positioned exactly at the inflow of the thoracic duct. On day 19 postoperatively, local thrombolysis with low-dose recombinant tissue plasminogen activator was started and administered for 26 h and 27 min, with simultaneous low-dose unfractionated heparin infusion as per our institutional thrombolysis protocol. Due to increased bleeding from the thoracic drains, recombinant tissue plasminogen activator was then paused and again restarted on day 21. The total duration of low-dose recombinant tissue plasminogen activator for local thrombolysis was 39 h and 30 min.

Focal follow-up ultrasound of the thoracic duct–venous junction was performed every 8 h. Follow-up of all thrombosed regions supra- and infra-diaphragmatically and transcranial ultrasound to exclude brain hemorrhage due to thrombolysis was performed daily.

Follow-up ultrasound examinations under local thrombolysis demonstrated initially progredient disaggregation and then gradual decrease of residual thrombogenic material at the thoracic duct–venous junction. A significant reduction in thrombus volume was found both supra- and infra-diaphragmatically. No cerebral secondary complication due to thrombolysis was identified.

**Fig. 3:**
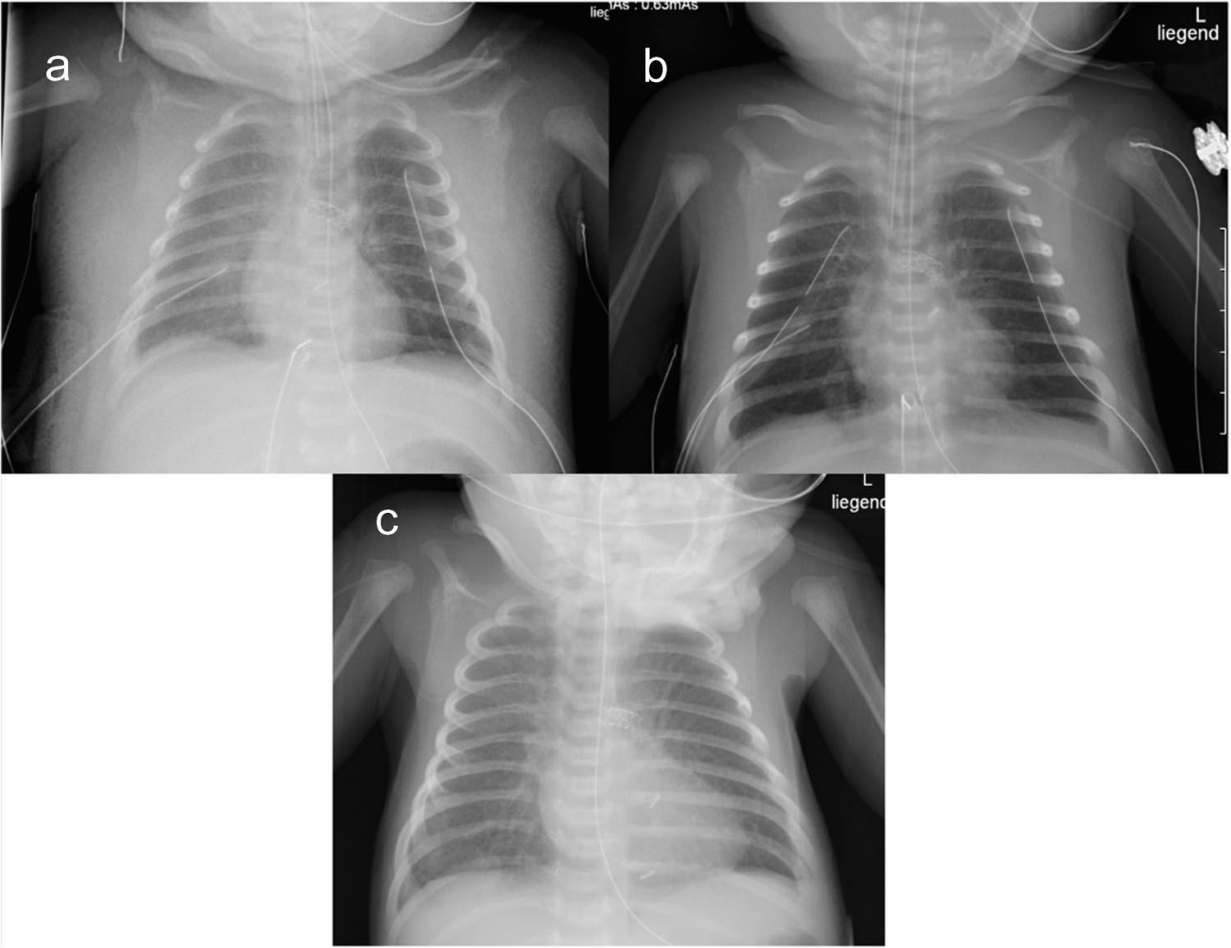
Serial chest radiographs in a 3-kg female neonate with a high-output chylothorax. **a** Day 16 of life: 1 day after percutaneous catheter intervention. Anteroposterior chest radiograph shows anasarca, pulmonary edema, and a right-sided pleural effusion. The patient is intubated, and thoracic and pericardial drains are in place. **b** Day 23 of life: 4 days after percutaneous thoracic duct access and mechanical thrombus clearance. Chest radiograph in anteroposterior projection shows resolving anasarca, pulmonary edema, and pleura effusion. Thoracic and pericardial drains remain in place. **c** Day 31 of life: 12 days post-intervention: Anteroposterior chest radiograph shows further improvement in anasarca. The child is extubated; thoracic and pericardial drains have been removed

A gradual decrease of the chylothorax from about 200 ml/kg to 3 ml/kg bodyweight of chyle per day was observed during the week after intervention (Figs. 1 and 3). Ultimately, the child stabilized, the thoracic drains could be removed, and the patient was extubated, transferred to the intermediate care unit, and able to be discharged home in good condition at 7 weeks of age.

## Discussion

After the initial diagnosis of extensive venous thrombosis with associated SVC syndrome, cardiac catheterization was performed to aspirate thrombotic material, and residual thrombus was compressed and pushed to the vessel walls during transluminal angioplasty. This is likely to have resulted in pushing thrombus into the thoracic duct at the thoracic duct–venous junction, which we observed on ultrasound. In this case, the thoracic duct obstruction was the cause of the high-output chylothorax. Patency of the thoracic duct and orthograde flow of chyle into the venous system was restituted after mechanical clearance of the thrombus from the thoracic duct–venous junction, enabling resolution of the chylothorax. Local lysis through the venous catheter positioned exactly at the thoracic duct–venous junction probably aided in preventing re-thrombosis.

Due to significant bleeding risks associated with the postoperative pericardial drain still being in place and the presence of a previous subacute brain hemorrhage/-infarction, local lysis was initially administered only for a short duration of time and with a limitation of dosage.

This case shows that in a 3-kg neonate with ultrasound guidance, it is not only possible to find but also to percutaneously access the thoracic duct by direct punction at the thoracic duct–venous junction. The procedure itself was not associated with any complications, was not time-consuming, and was without high radiation exposure. It effectively cured a life-threatening chylothorax without major complications.

We wish to highlight that thoracic duct occlusion should be considered a possible etiology of postoperative chylothorax following cardiothoracic surgery for congenital malformations. In such cases, the thoracic duct–venous junction should not only be examined with ultrasound, but if occluded, a percutaneous transluminal angioplasty of the thoracic duct performed by an interventional radiologist should be considered the primary treatment. Systemic thrombolysis could thereby possibly be avoided, or the dosage reduced, reducing the risk of adverse events such as intracranial hemorrhage in this vulnerable patient group.

## Data Availability

No datasets were generated or analysed during the current study.
